# Topical application of medicinal plant oils in pediatric-related disorders: A comparative review article based on traditional Persian medicine

**DOI:** 10.22038/AJP.2024.24303

**Published:** 2025

**Authors:** Maryam Mohammadian-Dameski, AhmadShah Farhat, Maliheh Motavasselian, Vahid Reza Askari

**Affiliations:** 1 *Department of Persian Medicine, School of Persian and Complementary Medicine, Mashhad University of Medical Sciences, Mashhad, Iran*; 2 *Neonatal Research Center, Department of Pediatrics, Faculty of Medicine, Mashhad University of Medical Sciences, Mashhad, Iran*; 3 *Applied Biomedical Research Center, Mashhad University of Medical Sciences, Mashhad, Iran*; 4 *Pharmacological Research Center of Medicinal Plants, Mashhad University of Medical Sciences, Mashhad, Iran *

**Keywords:** Anointment, Traditional Persian medicine, Medicinal oil, Pediatric diseases

## Abstract

**Objective:**

Researchers have considered medicinal oils to prevent and treat pediatric diseases. In the traditional Persian medicine (TPM) doctrine, anointment is widely used in order to avoid and treat diseases. This study aimed to evaluate and reconcile the uses of anointment in children in TPM and the new studies.

**Materials and Methods:**

Accordingly, TPM documents were scrutinized for anointments and their applications in pediatric disorders. Moreover, new studies were reviewed in Google scholar, PubMed, Medline, Scopus, and Web of Science until June 2022.

**Results:**

In the health preservation field, TPM scholars have recommended daily anointment with some oils like sweet almonds in the early months after birth to improve growth, strengthen the body, and prevent dry skin, and new studies have confirmed the effectiveness of some oils accordingly. In the treatment field, various oils are recommended, namely sweet almond oil for weight disorders, violet oil for insomnia, olive oil and chamomile oil for functional constipation, infantile colic and enuresis, and olive oil for diaper rash and atopic dermatitis and new studies have shown their effectiveness. Sweet almond, chamomile, violet, olive, and rose oils are among the most widely used oils in Persian medicine for children, which we have discussed in this study.

**Conclusion:**

Due to the non-invasive nature of anointment and the observation of minimal adverse effects in studies, it can be given attention for maintaining pediatric health and treating their diseases.

## Introduction

Infants and children are the most vulnerable group in society, and taking care of them is particularly important for a healthy future community (Abbasi et al., 2017; Baradaran Rahimi et al., 2020; Hamidnia et al., 2018). In conventional medicine, oral and sometimes invasive treatments, such as intravenous injections, are usually used, and less attention is paid to topical and non-invasive treatments. However, due to the delicacy of pediatrics, it is necessary to refer to topical and non-invasive treatments. Topical medications, including oils has been used on the skin for thousands of years in ancient civilizations to treat diseases. Topical drug administration reduces systemic drug toxicities (Abbasi et al., 2017; Baradaran Rahimi et al., 2021). Furthermore, topical application of a drug eliminates the hepatic first pass effects and other gastrointestinal (GI) influences, such as pH sensitivity and gastric emptying time causing a premature breakdown and metabolism of the medicine. In addition, patient compliance is increased, and systemic adverse effects are reduced (Abbasi et al., 2017; Tafti et al., 2017).

In the doctrine of traditional Persian medicine, anointment has an essential role in preventing and treating diseases, especially in infants and children (Hamedi et al., 2013; Hamidnia et al., 2018). Anointment, called aromatherapy (aroma means odor) in complementary medicine, is mainly done using herbs' aroma, which is different from anointing in Persian medicine. In Indian traditional medicine (Ayurveda), oils are used topically in various ways. For example, whole-body massage which is usually done with sesame oil, coconut oil, and almond oil, in addition to treatment , is also done to maintain health (Ali et al., 2015; Tavakoli-Dastjerdi et al., 2019).

But, of course, it is worth noting that in Persian medicine, anointing has an auxiliary role in prevention and treatment and should be done along with lifestyle modification, including diet, sleep, physical activity, and psychological issues.(Ghazanfari et al., 2022; Tavakoli-Dastjerdi et al., 2019). In recent years, this treatment method has attracted the attention of researchers and has been studied in the form of clinical trials in various fields (Baradaran Rahimi et al., 2021; Baradaran Rahimi et al., 2020). Among the most widely used pediatric oils in Persian medicine are olive, sweet almond, sesame, violet, chamomile, and red rose oils (Baradaran Rahimi et al., 2021; Ghazanfari et al., 2022; Hamedi et al., 2013). 

In the present study, we intend to explain the applications of anointment in children in traditional Persian medicine documents and match the evidence of new clinical studies.

## Materials and Methods

Traditional Persian medicine books and documents were scrutinized for anointments and their applications in pediatric disorders. Authoritative sources of traditional Persian medicine were searched in the comprehensive software of *Tibb-e Noor*. Contextually, Persian medical books such as *Eksir-e A'zam* and* Tibb-e Akbari*, as well as Persian pharmaceutical books such as* Makhzan al-adviah*, were reviewed to find the topical uses of oils in treating diseases. Moreover, new findings and studies were searched and collected by the keywords, including oil, topical, massage, neonates, infants, children, pediatric, and in combination with the scientific name of high-consumption medicinal oils in Google scholar, PubMed, MEDLINE, Scopus and Web of Science until June 2022. All Persian and English studies were considered for reporting therapeutic oils' effectiveness, safety, and toxicity. 

## Results

### Topical use of oils in health preservation management (hefz-al-sehheh, hygiene) of children

**Traditional persian medicine**

Daily anointment for boys up to four months and girls up to two months, especially after bathing, helps to strengthen the body and moisturize the skin. To relieve fatigue and muscle stiffness in infants, anointment and massage of both sides of the vertebrae from the back to the neck can be considered. To care for the umbilical cord, it has been recommended that after cutting the umbilical cord, a cloth soaked in olive (*Olea europaea* L.) or sesame (*Sesamum orientale* L.) oil is placed on it and repeat the procedure until it falls off. 

### Recent studies

In Turkey, one of the traditional methods of umbilical cord care is the use of olive oil. In a 2010 study in Turkey on infants' umbilical cords, they were divided into a control (keeping the umbilical cord dry) and a study group (lubricating the umbilical cord with olive oil). Ultimately, they observed that the average time for umbilical cord separation was not significantly different between the two groups. Still, in most of the infants in the study group, the umbilical cord separated before ten days, which was a significant difference here. The researchers also prepared bacteria cultures from the neonates' umbilical cords at three different times, which were not significantly different between the two groups. They concluded that olive oil could be used to care for the neonatal umbilical cord (Erenel et al., 2010).

### Topical use of oils in the treatment of pediatric diseases

**Nutritional and weight gain problems**

**Traditional persian medicine**

For the treatment of weight loss due to dryness of mizaj (with symptoms such as insomnia and dry skin), anointing the body with moisturizing oils such as violet, pumpkin (*Cucurbita* spp.), or almond oil (Aghili, 2006) after a bath with a moderate temperature and a short time are recommended (Table 1). Furthermore, for weight loss due to poor absorption of food by organs (mainly due to increased quality of cold in the body, which is characterized by symptoms such as coldness in touch and slowness of movement), anointing of the body with olive oil is highly recommended (Nazem Jahan, 2008). 

### Recent studies

Recently, it has been shown that the massage of premature infants with anointment increases their weight and height. In fact, this is because the skin of premature infants, due to its thinner and more vascular tissue, absorbs high amounts of fatty acids and consequently receives more calories (Panigrahi et al., 2016).

Furthermore, Safdarian et al. in their study of preterm infants without gastrointestinal nutrition, showed that massaging the body of these infants with sunflower (*Helianthus annuus*) oil can eliminate the deficiency of essential fatty acids in the serum and its clinical complications and replace intravenous lipids that have potentially serious and dangerous adverse effects (Safdarian and Hosseini, 2009). In a study in India, researchers added nano-micronutrient liposomes (e.g., iron, folate, vitamin B12, and vitamin D) to infant body oil and used them, which caused the supply of micronutrients (Apte et al., 2021). Moreover, a systematic review showed that topical application of coconut (*Cocos nucifera*) oil could reduce the risk of infection, increase weight and improve skin condition in preterm infants (Pupala et al., 2019). In line with the previous findings, the study of Alizadeh et al. showed that massaging the body of premature infants with sunflower oil, even for five days, can significantly increase the weight of these infants and reduce their hospital stay (Alizadeh P, 2013). However, the control group did not have an intervention; it has not been elucidated whether this therapeutic effect can result in a combination of massage and sunflower oil (Alizadeh et al., 2013). In another study, body massing of preterm infants with sunflower oil reduced the incidence of nosocomial infections (Darmstadt et al., 2004).

Additionally, two studies have examined the effect of sweet almond oil on preterm infants. First is the study of Caglar et al. which showed that massaging the baby's body with sweet almond oil increases stratum corneum hydration and improves their skin condition (Caglar et al., 2020). The second is the Douret study Et al. in which the use of sweet almond oil combined with sensory-motor stimulation massage for healthy preterm infants resulted in weight gain, nerve growth, and shorter hospital stay (Table 2) (Vaivre‐Douret et al., 2009). Therefore, neonatal body massage using appropriate oil strengthens the tissue and function of the skin as the body's first defense barrier and systematically strengthens the immune system's function, reduces transepidermal water loss, improves thermal regulation and reduces transepidermal water loss, improves thermal regulation and the risk of nosocomial infections (Panigrahi et al., 2016). 

### Pain and inflammation of the gums caused by teething

**Traditional persian medicine**

Anointing of gum with sweet almond oil, chamomile (*Matricaria recutita* L- *Chamaemelum nobile* L.) oil, or olive oil, rubbing the head and neck with a mixture of violet oil and lukewarm water, dripping lukewarm violet oil in the ear on the same side, topical application of nightshade extract with lukewarm of rose (*Rosa gallica* L - *Rosa centifolia* L) oil on the jaw is recommended (Table 1). (Razi, 2000; Shaharzani, 2007).

### Recent studies

Currently, in complementary medicine, the use of ointments based on tea tree (*Melaleuca alternifolia*) oil, clove (*Eugenia caryophyllata*) oil, and olive oil is highly recommended (Markman, 2009; Tsang, 2010). 

### Excessive crying and insomnia

**Traditional persian medicine**

It has been used and documented that rubbing violet, lotus, squash, almonds, or rose oil on the head, soles of the feet, navel, and anus or dripping them into the nose to treat insomnia (Nazem Jahan, 2008). Apart from rose oil, these oils have a cold and wet nature, modulate the temperature, and moisturize the organs (Aghili, 2009). In addition, in severe infants' crying, for any reason that does not respond to other treatments, poppy seed oil or lettuce (*Lactuca sativa*) seed oil can be used for the anointing of the head and both sides of the dorsal vertebrae (Table 1). (Baha'-al-doleh, 2003).

### Recent studies

In the study of Ranjbar et al. improvement in sleep disorder in children was observed by rubbing lettuce seed oil on the forehead and temporal areas (Ranjbar et al., 2020). The study by Feyzabadi et al. also showed that intranasal consumption of violet oil could become effective in patients with chronic insomnia (Feyzabadi et al., 2018).

### Headache

**Traditional persian medicine**

In some types of headaches, oils can be used topically, depending on the cause of the headache. The recommended oil type is different and depends on the nature and temperament of the headaches and patients, respectively. For example, rose, violet, lotus, or willow oil with rosewater and vinegar could be used for hot types. In contrast, for cold types, lily oil, Jasminum, Marjoram, and Chamomile are suggested. As regards headaches due to dryness, rubbing moisturizing oils such as almond, sesame, violet, pumpkin, and lotus oils on the head, soles of the feet, umbilicus, anus, and distilling in the nose and ears are proposed (Table 1). Concerning headaches due to brain weakness and high steam, rose oil and chamomile oil have been offered, respectively (Shaharzani, 2007).

### Recent studies

In a study by Zargaran et al. on patients suffering from migraine headaches, topical application of chamomile oil reduced pain, nausea, vomiting, photophobia, and phonophobia after 30 minutes (Zargaran et al., 2018). In another study, researchers observed that topical application of rose oil in patients with migraine headaches, reduced pain intensity in the hot type after 30, 45, 60, 90, and 120 minutes (Niazi et al., 2017). In Ahmadifard's study, the topical application of basil essential oil three times a day for three months reduced the pain intensity and the frequency of migraine attacks (Ahmadifard et al., 2020). Furthermore, a review study showed that topical application of peppermint essential oil has a proven effect in the treatment of acute tension headaches, and its 10% solution can be used in adults and children over six years (Göbel et al., 2016).

### Cough

**Traditional persian medicine**

The type of oil consumed depends on the cause of the cough origin. For example, in children, coughing is often caused by excessive moisture and sometimes by dryness in the lungs and airways. Therefore, for coughs due to thick moisture, a mixed formulation of wax with almond oil with honey, and for coughs caused by dry throat and chest, rub a mixture of wax and moisturizing oils such as sweet almond oil and violet oil on the chest and neck is useful (Aghili, 2009; Nazem Jahan, 2008).

### Recent studies

No recent studies have proven the application of anointment of oils for preventing and improving the infant's cough yet.

### Muscle cramps

**Traditional persian medicine**

In muscle cramps due to the accumulation of waste products, after cleansing, it has been recommended that you can use warm oils such as bitter qust (*Saussurea costus*), Rue (*Ruta graveolens* L), and jasmine. However, moisturizing oils such as violet, pumpkin, and sweet almond can be used for cramps due to dryness. Chamomile oil is also helpful for relieving cramps caused by wind (gas). 

### Functional indigestion

**Traditional persian medicine**

If indigestion is due to cold, mastic (*Pistacia lentiscus*) and lily oil, and if it is due to cold and moisture, lily oil and bitter qost will be useful (Shaharzani, 2007).

### Recent studies

In a study of patients with functional indigestion, gastric massage with mastic oil three times a day for four weeks was compared with sesame oil. Both groups had improvement in all four symptoms, namely premature satiety, postprandial heaviness, epigastric pain, and epigastric burning. Still, the reduction in the severity of premature satiety in the mastic group was significantly more than in the control group. In addition, satisfaction with treatment in the mastic group was considerably higher than in the sesame group (Baradaran Sattarzadeh et al., 2021).

### Functional constipation

**Traditional Persian Medicine**

Here, the type of oil used depends on the cause of constipation. In most cases of children, constipation is caused by excess moisture in the intestinal wall (with symptoms of stool stiffness and decreased feeling of the need to defecate and sluggish bowel movements) and abdominal massage with a combination of olive oil with warm water is recommended (Nazem Jahan, 2008). Also, abdominal anointing with violet oil is used for constipation caused by heat and dryness (with symptoms of fragmented and bullet-shaped stools) (Shaharzani, 2007).

### Recent studies

In a study, abdominal massage with an ointment containing olive oil improved constipation in children aged 1 to 4 years (Table 2) (Arman-Asl et al., 2021). In another study, an olive oil enema was performed on severe constipation in children, which was able to reduce constipation by more than 76% (Yokoi and Kamata, 2021).

### Infantile colic

**Traditional persian medicine**

The cause of colic in most infants is stomach weakness, drinking excessive milk, or unsuitable quality breast milk, which causes bloating and pain in the intestines. In these conditions, massage of the back (spine) and abdomen with rose oil, rubbing chamomile, lily, fennel (*Foeniculum vulgare*), mastic, and olive oil are recommended (Nazem Jahan, 2008).

### Recent studies

In several studies, the effect of different oils with or without massage on the symptoms of infantile colic has been studied, and each had a pleasing effect. They include massaging the whole body with olive oil three times a day (Huhtala et al., 2000), massaging around the umbilicus with chamomile oil three times a day at rest (Salehipoor et al., 2019), or rubbing chamomile oil on the abdomen and sides of the baby without massage six times a day at rest (Sorme et al., 2019) and anointment of the abdomen with lavender (*Lavandula angustifolia, Lavandula stoechas*) oil at the time of onset of the attack (Table 2) (Çetinkaya and Başbakkal, 2012).

### Enuresis and urinary incontinence

**Traditional persian medicine**

Applying turpentine, lily, chamomile, and narcissus (with symptoms of cold in touch and light urine) in enuresis and incontinence due to cold and humidity (*Narcissus tazetta* L.) oils are proposed. However, rose oil is used in urinary incontinence due to heat (with symptoms of yellow and dark urine) (Nazem Jahan, 2008).

### Recent studies

Three studies investigated the effect of bladder anointing with chamomile oil, olive oil, and qost oil in children with symptomatic urinary incontinence, which were significantly different compared with placebo and reduced the symptoms (Ilkhani et al., 2021; Lestariningsih and Triwijayanti, 2020; Sharifi et al., 2017).

### Dermatitis caused by diapers

**Traditional persian medicine**

Rose oil is useful for groin ulcers caused by diapers, and if there is a lot of dryness in the area, violet oil is functional (Shaharzani, 2007).

### Recent studies

A multicenter open-label trial of 1-36 months infants with a known history of recurrent diaper dermatitis reported that almond oil-based ointment conferred a protective effect against future episodes of diaper dermatitis, with 90% of the subjects showing a decrease in frequency or total absence of diaper dermatitis after using the ointment (Lio, 2016). In another study, researchers found that a product containing honey, beeswax, olive oil, and propolis more substantially improved the symptoms of infants with diaper rash than nystatin cream (Al-Waili, 2005). In this regard, Sharifi et al., in their study, observed a similar effect of 1.5% olive oil ointment and 1.5% calendula (*Calendula officinalis- Calendula persica*) ointment on diaper dermatitis (Sharifi‐Heris et al., 2018).

### Atopic dermatitis

**Traditional persian medicine**

For inflammatory skin lesions similar to dermatitis, rubbing wheat (*Triticum aestivum*) oil and chicken or duck fat in combination with tragacanth or *Aloe vera* (or *Aloe barbadensis* miller) is recommended to reduce the inflammation (Shaharzani, 2007).

### Recent studies

In a study in 2008, 173 premature infants were divided into three groups: regular cream, a cream containing olive oil, and a control group, and they were oiled daily for one month. In the end, the olive oil group infants had less dermatitis than the other groups, and both oiled groups had better skin conditions (Kiechl‐Kohlendorfer et al., 2008). In addition, one study found that creams containing 20% ​​improved on improving the symptoms of children with atopic dermatitis than regular moisturizers (Dwiyana et al., 2019).

### Skin rash

**Traditional persian medicine**

For the treatment of mild to moderate rashes, anointment with oils such as rose oil and myrtle oil that strengthen the tissue is recommended (Shaharzani, 2007).

### Recent studies

Tea tree oil (Markum and Baillie, 2012) and Australian lemon (*Citrus australasica*) essential oil (Burke et al., 2004) have been effective in children with *Molluscum contagiosum*.

### Wound

**Traditional persian medicine**

Sesame oil, rose and henna were reported to be helpful in wound healing (Shaharzani, 2007).

### Recent studies

New studies in this field include: Accelerating the healing of skin wounds with essential oil of rose (20% and 25%) (Dehghani et al., 2015).

### Overview of the therapeutic properties of some of the most widely used oils from the perspective of conventional medicine

**Sweet almond oil**

Sweet almond oil is a rich source of vitamin E and B groups of vitamins, amino acids, minerals, and 26% of carbohydrates and is used in skin softening and cosmetics (Varaei, 2019). Other components of almond oil include tocopherols, tocotrienols, polyphenols, phytosterols, and volatile organic compounds. This oil has a hypnotic effect that is probably related to amino acids such as glycine, glutamine, arginine, and ornithine. Its anti-oxidant effect is attributed to tocopherols, and its anti-inflammatory effect relies on polyphenols, flavonoids, and monounsaturated fatty acids. In addition, this oil has been studied to improve skin conditions and weight gain in premature infants and prevent the recurrence of diaper dermatitis (Karimi et al., 2021; Ouzir et al., 2021).

### Chamomile oil

Chamomile plant has two different species: German chamomile (*Matricaria recutita* L.) and Roman chamomile (*Chamaemelum Nobile* L.). Both contain similar compounds and therapeutic effects, including sesquiterpene (such as bisabolol and farnesene), sesquiterpene lactones (such as chamazulene, matricin), flavonoids (such as apigenin, luteolin), and volatile oils (Sharafzadeh and Alizadeh, 2011). In addition, lipophilic compounds such as chinazolin, matrixin, bisabolol, and its oxides have pronounced anti-inflammatory effects, and hydrophilic compounds such as flavonoids and coumarins have strong anti-spasmodic effects. In general, chamomile is useful in healing wounds and some skin diseases such as psoriasis, eczema, and acne due to its anti-inflammatory, anti-microbial and anti-oxidant effects. It has also been shown that this plant has analgesic effects (Dashti Rahmatabadi et al., 2001).

### Violet oil

The violet plant contains various compounds, including glycosides, tannins, flavonoids, saponins, alkaloids, mucilage, vitamin C, salicylic acid, coumarin, violin, and a small amount of melatonin. Various studies have studied and confirmed the anti-inflammatory and hypnotic effects of violet. Its phytosterol also reduces erythema, itching, and inflammation of the skin. In addition, this plant has analgesic, antifungal, anti-oxidant, diuretic, and antipyretic effects in modern herbal medicine. Hence, its topical use is helpful for improving itching, urticaria, burns, inflammatory conditions, and insomnia (Feyzabadi et al., 2018; Khorsand et al., 2019; Tafazoli et al., 2020; Zojaji, 2015).

### Olive oil

The main constituents of olive oil include oleic acid, phenolic components, and squalene. Studies have shown that the phenolic compounds of olive oil have beneficial effects on some physiological indicators such as oxidative stress, inflammatory markers, cellular function, and anti-microbial activity (Ezzeddin et al., 2015). Oleocanthal is a phenolic compound in extra virgin olive oil that has anti-inflammatory and analgesic properties and, like ibuprofen, inhibits cyclooxygenase enzymes in the synthesis of prostaglandins (Beauchamp et al., 2005). There are at least 30 phenolic compounds in olive oil with potent anti-oxidant properties (Tuck and Hayball, 2002). Olive oil can also be used topically to treat skin problems such as psoriasis. Topical and daily use of this oil has been reported to improve skin problems in premature infants, which may reduce the risk of skin infections (Varaei, 2019).

### Rossa damascene oil

Rose and its derivatives contain phenethyl alcohol, citronellol, linalool, and geraniol, which have inflammatory, analgesic, anti-oxidant, anti-cancer, anti-microbial, antianxiety, antidepressant, and muscle relaxant effects (Sadeghi Avval Shahr et al., 2014).

Some contraindications or precautions for topical use of oils in children from the perspective of traditional Persian medicine

In traditional Persian medicine, anointment is not recommended in some conditions. These include anointment of the upper head in patients with symptoms of runny nose and burning and thin post-nasal discharge (Shaharzani, 2007).

### Adverse effects in recent studies

A few studies have reported adverse effects, and most studies have been without adverse effects (Table 3). The most common adverse effects are irritation of the eyes, mucous membranes, and skin and hypersensitivity, especially to oils containing aldehydes and phenols, as well as photosensitivity to oils containing furocoumarins. Contact allergies are more likely to be caused by the oxidation of monoterpenes due to poor storage conditions (Ali et al., 2015). Inhaled essential oils are prohibited in patients with asthma and epilepsy (Harding, 2006). Pouring oil into infants' noses carries a risk of pneumonia and subsequent bronchiectasis (Niggemann and Grüber, 2003). 

## Discussion

Anointing in traditional Persian medicine is widely used for the prevention and treatment of diseases in various organs of the body (Abbasi et al., 2017). In the field of health protection, Persian medicine scholars, to improve growth, strengthen the body, and prevent dry skin, have recommended daily anointment in the early months of birth with oils such as violet or sweet almond oil. To improve weighing, oils such as violet, sweet almond, squash, and olive, are recommended depending on the cause and nature of the diseases. In this regard, recent studies have examined the effectiveness of some oils, such as coconut and sunflower oil, in weight gain, improving skin condition, and reducing nosocomial infections in premature infants, and their effectiveness has been confirmed. But the view of Persian medicine on anointment in infants is more comprehensive because it is not limited to premature or low birth weight infants and recommends that healthy infants strengthen the body and improve growth. However, more studies are needed to determine the preventive effect of this procedure on diseases and the developmental status of infants in the future. 

Also, In the field of treatment, Persian medicine for sleep disorders and some respiratory, gastrointestinal, urinary, muscular, and skin diseases find anointment with special oils beneficial. In contrast, conventional medicine usually uses topical oils in the form of products such as creams and ointments and in the field of musculoskeletal diseases and skin diseases. New studies have paid more attention to these diseases. Clinical studies in infants and children have focused on skin problems such as atopic dermatitis, diaper rash, and skin rashes, often with a phytotherapy perspective (Baradaran Rahimi et al., 2020). A limited number of clinical studies have been conducted from the perspective of Persian medicine and have had acceptable results, such as the effect of lettuce seed oil on insomnia, olive oil on constipation, chamomile oil on infant colic, and olive and chamomile oil on nocturnal enuresis and urinary incontinence. 

The main difference between the new trials and whether they are recommended in traditional Persian medicine, the choice of drug is based on its nature and a causal approach (change of mizaj, debility, gas, etc.). Although most recent studies have taken the approach of phytotherapy or herbal medicine, there is a great need for clinical studies with a traditional medicine approach. The traditional medicine approach is expected to increase the efficiency of investigations. From the perspective of Persian medicine, it is important to pay attention to the child's temperament (mizaj) and the nature of the oil consumed to prevent complications as much as possible.

**Figure 1:**
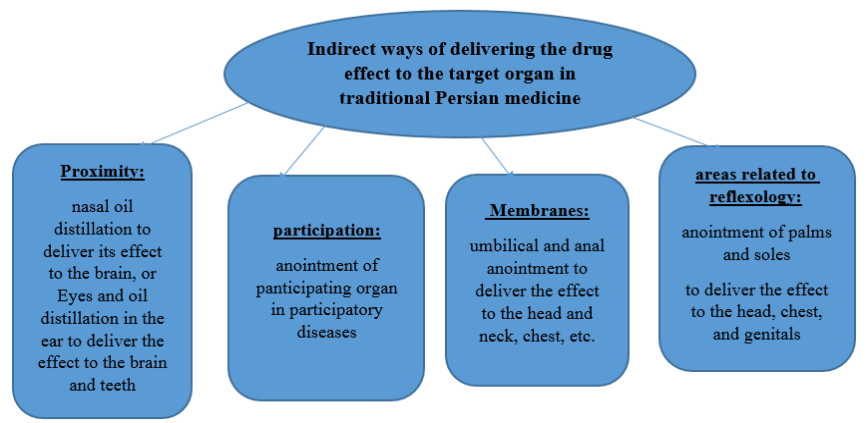
Various indirect ways to deliver the drug effect to the target organ, such as reflexology, proximity, membranes, and participation.

Another difference is that in traditional medicine sources, there are various indirect ways to deliver the drug effect to the target organ, such as reflexology (anointment of palms and soles), proximity (nasal oil distillation to deliver its effect to the brain, or Eyes and oil distillation in the ear to deliver the effect to the brain and teeth), through membranes (umbilical and anal anointment to deliver the effect to the head and neck, chest, etc.), through participation (anointment of participating organ in participatory diseases), while in conventional medicine oils are mainly used for direct topical use, and only aromatherapy in complementary medicine is considered as an indirect way (Figure 1).

**Table 1:** Commonly oils are used as topical applications for the prevention and treatment of children in traditional Persian medicine

oil name	Persian medicine
Sweet almond oil	health protection: massaging girl neonates treatment: Insomnia, teething pain in infants, dry cough, dry skin, dry nose, hair loss due to dry skin, and thinning due to dryness of mizaj (Aghili, 2006, 2009; Ibn-e Elyas, 2003; Nazem Jahan, 2008; Shaharzani, 2007; yousefi heravi, 2004)
Chamomile oil	treatment: swelling from cold, teething pain in infants, muscle and visceral pain, hair loss due to narrow pores or dry skin, runny nose and thick post-nasal discharge, cold headache, urinary retention, urinary incontinence from cold, and laxity of the bladder (Aghili, 2009; Ibn-e Elyas, 2003; Ibn-e Nafiss, 2008; Nazem Jahan, 2008)
Violet oil	health protection: massaging girl neonates, nail health protection treatment: insomnia, teething pain in infants, dry cough, hair loss due to lack of nutrients, dry organs and joints, headache, dry skin, dry nose, runny nose, and burning and thin post nasal discharge (Aghili, 2006, 2009; Ibn-e Elyas, 2003; Nazem Jahan, 2008)
Olive oil	health protection: umbilical cord care treatment: tonify gums, teeth, and other organs, reduce pain, teething pain in infants, wound healing, laxative, elimination of infection, treatment of seborrhea and scabies, weight loss due to poor food absorption by organs (Aghili, 2006, 2009; Varaei S, 2019)
*Rossa damascene* oil:	Treatment: tonify organs, teething pain in infants, pain relief, insomnia, mouth ulcers, and nausea (Aghili, 2006, 2009; Ibn-e Sina, 2005)

**Table 2:** New studies on the topical use of oils

**Oil name**	**Authors and year of publication**	**Application and result**	**Oil dose and duration of use**	**Compared to**	**Type of oil**	**number of patients enrolled in treatment and placebo groups**
Sweet almond (*Amygdalus communis* - *Prunus communis*)	(Vaivre‐Douret et al., 2009)	enhanced weight gain and neurological development, and a shorter stay in the hospital in both groups of oils (almond and ISIO4)	15 min, twice per day, For 10 days, Mix with a vegetable emollient	ISIO4 oil (the combination of 4 seed oils: rapeseed oil, extra virgin olive oil, sunflower oil, grape seed oil), placebo and control	Not mentioned	almond oil (n=12) ISIO4 oil (n=12) placebo (n=12) control (n=13)
	(Caglar et al., 2020)	better stratum corneum hydration in the sunflower seed oil (SSO) and almond oil (AO) groups than in the control group, no difference between the SSO and AO groups, without harmful effects on the skin	1 mL/kg for 2 to 3 minutes, 4 times a day for 5 days to the whole body	Sunflower oil and control group	Cold pressed	Almond oil (n=30) Sunflower seed oil (n=30) control (n=30)
	(Lio, 2016)	no erythema, decrease in skin dryness, roughness, and an increase in skin suppleness, 90% decrease in frequency or total absence of diaper dermatitis	the almond oil-based ointment was used daily after each diaper change over 28 days	Pre-test post-test	-	n= 60
*Aloe vera* (*Aloe barbadensis* miller)	(Ghanipour Badelbuu et al., 2019)	no difference between the Aloe Vera, chamomile, and routine treatments	a layer of 95% Aloe Vera ointment that covers the lesion, TDS, with routine treatment (a mixture of zinc oxide, hydrocortisone, and Clotrimazole) for 6 days	routine treatment alone and routine treatment with chamomile ointment	-	Chamomile (n=30), Aloe Vera (n=30), and control group (n=28)
Basil (*Ocimum basilicum* L.	(Ahmadifard et al., 2020)	Reduce the severity and frequency of migraine attacks at higher doses	every 8 hr to the frontal and temporal areas for 3 months	2, 4, and 6% concentrations and placebo group	essential oil	Basil 2% (n=36) Basil 4% (n=36) Basil 6% (n=34) Placebo (n=35)
Calendula (*Calendula officinalis*, *Calendula persica*)	(Sharifi‐Heris et al., 2018)	both olive ointment and calendula ointment provide the same results in the healing of Diaper dermatitis	1.5% calendula ointment after diaper changing per day for a period of 7 days	1.5% olive ointment	Not mentioned	olive ointment (n=37) calendula ointment (*n*=39)
German chamomile (*Matricaria recutita* L.) Roman chamomile (*Chamaemelum nobile* L.)	(Ghanipour Badelbuu et al., 2019)	no difference between the Aloe Vera, chamomile, and routine treatments	a layer of ointment that covers the lesion, TDS, with routine treatment (a mixture of zinc oxide, hydrocortisone, and Clotrimazole) for 6 days	routine treatment alone and routine treatment with aloe vera ointment	-	Chamomile (n=30), Aloe Vera (n=30), and control group (n=28)
	(Salehipoor et al., 2019)	improve and reduce the symptoms of infantile colic	five drops, three times a day for 14 days	paraffin	In sesame oil	Chamomile (n=34), Placebo (n=34)
	(Sorme et al., 2019)		5 to 7 drops, 6 times a day, on two areas of the body each time (back and abdomen) for 7 days	mineral oil	In mineral oil	Chamomile (n=51), Placebo (n=51)
	(Sharifi et al., 2017)	Decrease the frequency of nocturia in children with monosymptomatic nocturnal or daytime enuresis.	6 drops on the perineal and suprapubic area of children one time per night	sweet almond oil (placebo)	in sweet almond oil	Chamomile (n=40), Placebo (n=40)
	(Zargaran et al., 2018)	Reduction of pain, nausea, vomiting, photophobia, and phonophobia after 30 minutes in patients with migraine headache	2 ml of chamomile oil oleogel is applied topically on the forehead, temporal, and behind the ears when a headache is started. It will be done two times, after 2 weeks washing time the groups change their oil	placebo	oleogel prepared from chamomile oil (extraction of chamomile flower ingredients in sesame oil) in compression with placebo, including an oleogel prepared from 10% Chamomile oil and 90% liquid paraffin	drug-placebo (n=38) placebo-drug (n=34)
coconut (*Cocos nucifera*)	(Saeedi, GHOLAMI, Dinparvar, & Kabirian, 2011)	positive effect on weight gaining in preterm newborns	Four ml was massaged for 5 minutes, 4 times a day for 7 days	Only massage) and (Control)	Not mentioned	massage therapy with oil (n=33), massage therapy without oil (n=30) and control group (n=30)
Costus (*Saussurea costus* Lipsch.)	(Ilkhani et al., 2021)	effective in children with monosymptomatic nocturnal enuresis with a 74.5% response rate	20 drops twice a day topically (in the morning and before sleep) on the area between the navel and pubic region without massage for 4 weeks.	Cold pressed sesame oil as the control group	in sesame oil	Costus oil (n=39) Sesame oil (n=43)
Lavender *(Lavandula angustifolia,* *Lavandula stoechas*)	(Çetinkaya & Başbakkal, 2012)	effective for the reduction of infantile colic symptoms	1 drop of lavender oil mixed with 20 ml of almond oil, start the massage within 1–2 min of the onset of thecolic attack last between 5 and 15 min; for 4 weeks	control	In almond oil	Lavender oil (n=20) Control (n=20)
Australian lemon myrtle (*Backhousia citriodora*)	(Burke et al., 2004)	effective in reducing the number of lesions of molluscum.	one drop of 10% solution to each molluscum lesion once daily at bedtime for 21 days	vehicle (olive oil)	essential oil	active treatment (n=16) vehicle (n=15)
Lettuce (*Lactuca sativa* L.)	(Ranjbar et al., 2020)	significantly improved some of the most common sleep problems except snoring	7 drops on forehead and temporal areas for three weeks	topical placebo oil and clonidine capsule compared to lettuce seed oil and a placebo capsule	cold press	Lettuce oil (n=34) Control (n=33)
Mastic Oil (*Pistacia lentiscus*)	( Baradaran Sattarzadeh et al., 2021)	reduce early satiation better than the placebo, and satisfaction with the treatment was higher in the mastic group than the sesame group in patients with Functional dyspepsia (adults)	10 drops/TDS after meal) with massage for 4 weeks	sesame oil	In sesame oil	Mastic Oil (n=32) sesame oil (n=31)
Olive oil (*Olea europaea* L)	(Arman-Asl et al., 2021)	Effective in the treatment of children with functional constipation	85% olive oil ointment, twice a day, for 4 days	an ointment containing 85% liquid paraffin	Not mentioned	Olive oil ointment (n=20) placebo (n=20)
	(Yokoi & Kamata, 2021)	useful for more than three-quarters of children with severe chronic constipation	1-2 ml/kg, only one time either alone or followed several hours later by 1–2 ml/kg glycerin enemas once or twice a day	Pretest-posttest	Not mentioned	n=92
	(LESTARININGSIH & TRIWIJAYANTI, 2020)	decrease in enuresis in preschool children	Not mentioned	pretest and post-test	Not mentioned	n=32

	(Sharifi‐Heris et al., 2018)	both olive ointment and calendula ointment provide the same results in the healing of Diaper dermatitis	1.5% olive ointment, after diaper changing per day for a period of 7 days	1.5% calendula ointment	Not mentioned	olive ointment (n=37) calendula ointment (*n*=39)
	(Kiechl‐Kohlendorfer et al., 2008)	more effective than water-in-oil emollient in the prevention of dermatitis	a thin coat of the Olive oil cream (30% olive oil and 70% lanoline) on the body, except the face and scalp twice a day for 4 weeks	Bepanthen (a proprietary ointment including dexpanthenol and phenoxyethanol) and control group routine skincare	Not mentioned	Bepanthen (n=57) Olive oil (n=58) Control (n=58)
					
	(Nasiri, et al., 2015)	olive oil in combination with routine cares is more effective than routine cares alone on healing of diabetic foot ulcer, without any side effect	once a day for 4 weeks in both groups	control	Not mentioned	intervention group (n = 15), control group (n = 15)
Rose (*Rosa gallica* L - *Rosa centifolia* L)	(Niazi et al., 2017)	decrease the pain intensity In patients with hot migraine headaches (adults)	apply 2 ml of their prescribed oils on the forehead and temporal zones for 1 week,	Paraffin oil	In sesame oil (10 %)	Rose oil (n=15) placebo (n=14)
	(DEHGHANI et al., 2015)	Highest concentrations (20 and 25%) have promoted wound healing activity (rabbits)	Not mentioned	Not mentioned	essential oil of *Rosa damascena* extracted by Clevenger distillation	-
Sesame (*Sesamum indicum*)	(Shamloo et al., 2015)	reduce pain severity and frequency of received NSAIDs in patients with upper or lower extremities trauma (adults)	10 drops for each 50 square cm on the site of trauma, with massage, once a day until 10 days	control	manufactured by the Saman Sesame Oil Ltd.	Sesame oil (n=63) control (n=63)
Sunflower )*Helianthus annuus*(	(F. & AR, 2009)	Correction of triglyceride levels without the use of intravenous lipids in infants that do not receive nutrition through the gastrointestinal tract without adverse events	1 g/kg, TDS, for 7 days	control	Not mentioned	Sunflower oil (n=10) control (n=10)
	(Alizadeh P, 2013)	improve weight gain and shorten the length of NICU stay in premature neonates without adverse events	10 cc/kg/day divided three times per day, each session lasting 15 min with moderate pressure massage, for 5 days	control	Not mentioned	Intervention group (N = 22) Control group (N = 22)
	(Darmstadt et al., 2004)	improvement in skin condition and a highly significant reduction in the incidence of nosocomial infections without adverse events	4 g/kg/day divided three times per day for the first 14 day and two times per day for the second 14 days	control	Not mentioned	Intervention (n=51) Control (n=52)

	(Caglar et al., 2020)	better stratum corneum hydration in the SSO and AO groups than in the control group, no difference between the SSO and AO groups, without harmful effects on the skin	1 mL/kg for 2 to 3 minutes, 4 times a day for 5 days to the whole body	Sweet Almond oil and control group	cold extracted	Almond oil (n=30) Sunflower seed oil (n=30) control (n=30)
	(Dwiyana et al., 2019)	decreasing the transepidermal water loss score and improving scoring of atopic dermatitis index in children with mild atopic dermatitis, without adverse reactions	20% cream, twice daily, on both arms, both limbs, and in AD lesions (if present) For 28 days	a common commercial moisturiser	Not mentioned	Sunflower seed oil 20% (n=9) Control cream (n = 11)
Tea tree (*Melaleuca alternifolia*)	(Markum & Baillie, 2012)	The combination of essential oil of *M. alternifolia* (TTO) with organically bound iodine offers a safe therapeutic alternative in treating childhood molluscum.	twice daily for 30 days	TTO alone, combination of TTO and organically bound iodine (TTO-I), or iodine alone	essential oil	16 cases in each group
Sweet violet (*Viola odorata* L)	(Tafazoli et al., 2020)	Control of fever in febrile neutropenic children during the hospital course	20 drops, only one time	Placebo	In sweet almond oil	41 children divided into two groups.
	(Feyzabadi et al., 2018)	most influential among the three groups and more effective on sleep quality than sleep quantity (adults)	Intranasal dropping of Violet oil, Almond oil or placebo (1% solution of Carboxymethyl cellulose) in each nostril every night before sleep for 30 days	Sweet Almond oil and placebo	In sweet almond oil	Violet oil (n=22) Sweet almond oil (n=19) Placebo (n=19)
	(Saffar Shahroodi et al., 2019)	enhance tear production and improves tear film stability (adults)	2 drops 3 times a day for 1 month	Sweet Almond oil and placebo	In sweet almond oil	Violet oil (n=32) Sweet almond oil (n=29) Placebo (n=30)
	(Yazdi et al., 2020)	significant positive effects on the symptoms of allergic rhinitis (adults)	1 drop per nostril 2 times a day for 2 weeks	placebo	Ethanolic extract in sweet almond oil	Violet oil (n=38) Placebo (n=38)

**Table 3:** Mechanism of action and adverse effects of commonly used oils in children

**Oil name**	**Mechanism of action**	**Adverse effects**
**Sweet almond** **(** ***Amygdalus communis - Prunus communis*** **)**	**Sedative and hypnotic activity:** Decrease in the core body temperature, inhibitory actions of spinal cord interneurons, increases the production of GABA, stimulate detoxification of the liver (related to Amino acids like glutamine) amino acids such as glycine, glutamine, arginine, and ornithine can act as amino acid neurotransmitters; therefore, they play an important role in excitation and inhibition of synapses **Anti-inflammatory activity:** Inhibit the expression and formation of inflammatory mediators in macrophages (related to Polyphenols) and decrease serum E-selectin, reduce oxidative stress (associated with MUFAs, polyphenols, flavonoids) (Karimi et al., 2021) **Anti-oxidant effect**: scavenging free radicals by Tocopherols (Arranz, Cert, Pérez-Jiménez, Cert, & Saura-Calixto, 2008) and may be estimated by GSH, SOD, MDA, and CAT levels (Al-Attar, 2020)	atopic dermatitis rash and itching (Ouzir et al., 2021)
**German chamomile (** ***Matricaria recutita L*** **.)** **Roman chamomile (** ***Chamaemelum nobile*** ** L.)**	**anti-spasmodic effect:** by opening the K^+^ channels **anti-inflammatory effect:** inhibition of COX-2 and proinflammatory biomarkers in THP1 macrophages **neuroprotective effect:** reduction in NO levels (related to polyphenolic compounds [Flavonoides] including apigenin) (Zargaran et al., 2014)	contact dermatitis/skin reactions, eye irritation (when applied near the eyes), hypersensitivity reaction (Mollabashi, Ziaie, Bekhradi, & Khalesi, 2020) German chamomile: Contact dermatitis, Type I and IV allergy (Type IV in > 50% of Compositae-sensitive patients but low risk of sensitization/elicitation of dermatitis based on published reports), Immediate-type reactions from contact urticaria to more severe systemic reactions Roman chamomile: Contact dermatitis (Paulsen, 2002)
**Sweet violet** ** (*Viola odorata* L)**	**Anti-inflammatory effect:** Control serum levels of increased IgE, IL-4, IL13, eosinophilia, and increased mucosal secretion [19] **Anti-oxidant effect:** scavenge NO radicals and tyrosinase inhibition [20] **Hypnotic effects:** binding of flavonoids with high affinity to the benzodiazepine site of the GABA receptor and Rutin, a significant flavonoid component that has sedative effects in the brain, presence of melatonin in *V. odorata* flowers [21] **anti-microbial and antifungal activity**: monoterpenes and sesquiterpenes such as phenyl butanone, linalool, benzyl alcohol, α-cadinol, globulol, and viridiflorol **chronic cough, hoarseness, and stridor:** Mucilage with laxative properties[20]	There is not enough information to know if it is safe to put sweet violet on the skin. No serious adverse effects were reported in the studies
**Olive oil (** ***Olea europaea*** ** L)**	**Anti-inflammatory effect:** inhibition of the COX-1 and COX-2 enzymes in the prostaglandin-biosynthesis pathway, attenuating inflammatory mediators such as inducible NO synthase (by oleocanthal), inhibition of TNF-α (by oleuropein aglycone) (Beauchamp et al., 2005; Cicerale, Lucas, & Keast, 2012) **Anti-oxidant activity:** decrease reactive oxygen species (ROS) production and elicit significant free-radical scavenging effects, Reduce F2-isoprostane (a result of the free radical-induced peroxidation of arachidonic acid) levels (related to hydroxytyrosol and oleuropein) **Anti-microbial effect:** o-diphenol system of hydroxytyrosol, tyrosol, and oleuropein, scavenging of nitric oxide؟ (by hydroxytyrosol, oleuropein, and caffeic acid) (Tuck & Hayball, 2002)	detrimental impact on SC integrity and skin barrier function, and transepidermal water loss (Lin, Zhong, & Santiago, 2017) allergic contact dermatitis (Ernst, 2000)

**Rose (** ***Rosa gallica*** ** L - ** ***Rosa centifolia*** ** L)**	**Hypnotic** **activity:** affect the CNS through the olfactory way and trigger the release of some neurotransmitters such as dopamine and serotonin, stimulating the limbic system and suppressing the sympathetic system adrenaline level (Keyhanmehr et al., 2018) **Neuroprotective effect:** induces the neurite outgrowth and inhibits the Aβ fibrillization and deposition in the brain, inhibits apoptosis, promotes neuronal survival and synaptic plasticity, promotes cerebrovascular blood flow, angiogenesis, neurogenesis, and neuronal morphology Effect on length of dendrite similar to nerve growth factor (NGF) scavenging free radicals, and releasing neurotransmitter (acetylcholine), reduction of induced nitric oxide synthase (iNOS), nitric oxide-releasing, suppression inflammatory cytokines, such as TNF-α, IL-1β. by flavonoids, anti-oxidant functions. **Anti-oxidant functions:** donate hydrogen, scavenging reactive oxygen species (ROS), inhibiting lipid peroxidation, chelating metal ions, and other free radicals (Esfandiary et al., 2015) by flavonol glycosides including quercetin-3-O-glucoside, kaempferol-3-O-rhamnoside, and kaempferol-3-O-arabinoside **Analgesic effect:** effect on both phases centrally and peripherally, anti-oxidant activity (Hajhashemi, Ghannadi, & Hajiloo, 2010) **Anti-microbial activity:** citronellol, geraniol, and nerol **Anti-spasmodic effect:** through stimulating the b-adrenergic and opioid receptors and voltage-dependent calcium channels (Mahboubi, 2016)	Not mentioned

Considering the convenience, availability, and non-invasiveness of anointment for infants and children and also observing the minimum side effects and relative safety, it should be given more attention to health protection, improved growth, and treating diseases in infants and children. Furthermore, sweet almonds, chamomile, violet, olive, and rose oils are among the most widely used oils in Persian medicine for children. Therefore, we could suggest that Persian medicine experts teach healthcare workers and parents the use of anointment in infants and children so that they can use and benefit from this method in maintaining health and treatment along with other methods. Also, we could recommend that researchers choose some of the recommendations of traditional Persian medicine in the field of anointment in infants and children, which have not yet been clinically studied, for their subsequent studies.
